# Pancreatic-portal vein fistula in acute pancreatitis successfully treated with endoscopic approach

**DOI:** 10.1055/a-2299-2052

**Published:** 2024-04-25

**Authors:** Valborg Vang Poulsen, Annette Bøjer Jensen, Amer Hadi, Mia Prindahl Ærenlund, John Gásdal Karstensen, Srdan Novovic

**Affiliations:** 1 Pancreatitis Centre East, Gastrounit, Copenhagen University Hospital – Amager and Hvidovre, Copenhagen, Denmark; 2 Department of Radiology, Copenhagen University Hospital – Amager and Hvidovre, Hvidovre, Denmark; 3 Department of Clinical Medicine, University of Copenhagen, Copenhagen, Denmark

Acute pancreatitis is associated with numerous complications. Pancreatic-portal vein fistula (PPVF) is an exceptionally rare and diagnostically challenging example 1 2 . A 63-year-old man was admitted due to abdominal pain, weight loss, newly diagnosed diabetes, and elevated liver enzymes. Contrast-enhanced computed tomography revealed acute pancreatitis with fluid exudation and a necrotic collection in the head of the pancreas, accompanied by attenuation of fluid in the portal vein. Subsequent magnetic resonance cholangiopancreatography raised suspicion of PPVF.

Endoscopic retrograde cholangiopancreatography (ERCP) identified a stenosis in the pancreatic duct (PD) at the head of the pancreas, associated with an upstream fluid collection and a fistula into the portal vein. The PD was not visible as the contrast injection passed into the portal vein ( Video 1 ). The therapeutic intervention included pancreatic sphincterotomy with dilation of the PD stenosis with a 6-mm balloon catheter. Two 7 cm × 7 Fr double-pigtail stents were positioned within the fluid collection. The patient developed septicemia, which was treated with antibiotics. The patient was discharged after 45 days of hospitalization.

Endoscopic retrograde cholangiopancreatography and fluoroscopy with contrast and wire in the pancreatic fluid collection. Notice the contrast in the portal vein instead of the pancreatic duct.Video 1

At ERCP 2 months later, no communication between the PD and portal vein was evident, but the stenosis of the PD had recurred ( Fig. 1 ). The stenosis was dilated using a 4-mm balloon catheter, and one 7-Fr and one 5-Fr pancreatic stent were placed. The stents were replaced biannually during the following 12 months. There was no sign of PPVF, and the PD was patent, without stenosis. At 2.5 years’ follow-up, the patient was asymptomatic regarding acute pancreatitis and PPVF.

**Fig. 1:**
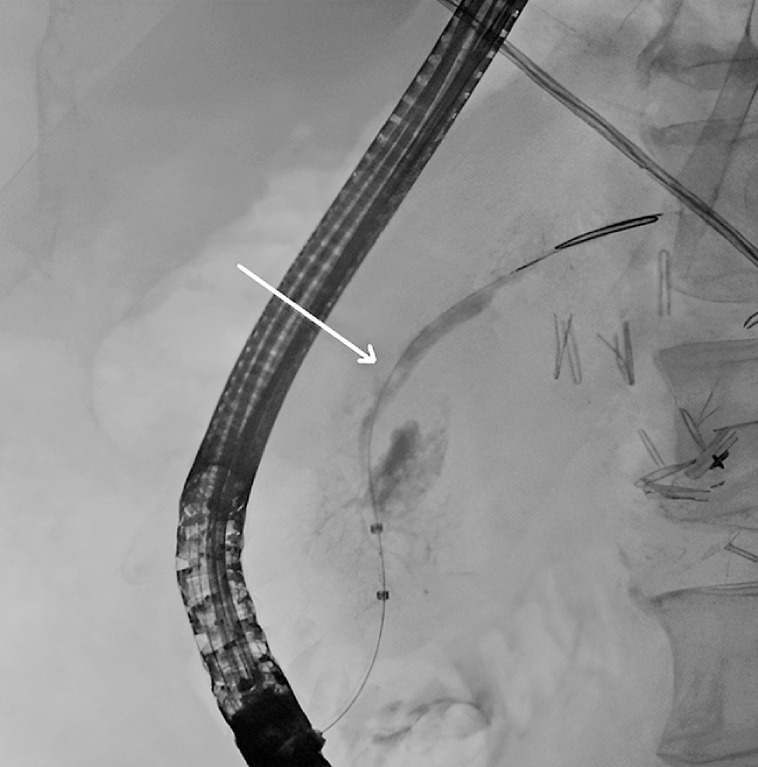
Endoscopic retrograde cholangiopancreatography 2 months after treatment of the pancreatic-portal vein fistula showed a stenosis in the pancreatic duct (arrow) and no contrast in the portal vein.

This case highlights the importance of recognizing PPVF as a potential complication of acute pancreatitis and not solely associated with chronic pancreatitis. The patient’s long-term survival underscores the significance of tailored interventions achieving favorable outcomes in complex complications of acute pancreatitis.

Endoscopy_UCTN_Code_CCL_1AZ_2AH

## Notes

Endoscopy E-Videos https://eref.thieme.de/e-videos
*E-Videos* is an open access online section of the journal *Endoscopy* , reporting on interesting cases and new techniques in gastroenterological endoscopy. All papers include a high-quality video and are published with a Creative Commons CC-BY license. Endoscopy E-Videos qualify for HINARI discounts and waivers and eligibility is automatically checked during the submission process. We grant 100% waivers to articles whose corresponding authors are based in Group A countries and 50% waivers to those who are based in Group B countries as classified by Research4Life (see: https://www.research4life.org/access/eligibility/ ). This section has its own submission website at https://mc.manuscriptcentral.com/e-videos .
